# Plasma cytokine levels characterize disease pathogenesis and treatment response in tuberculosis patients

**DOI:** 10.1007/s15010-022-01870-3

**Published:** 2022-06-27

**Authors:** Monika M. Vivekanandan, Ernest Adankwah, Wilfred Aniagyei, Isaac Acheampong, Augustine Yeboah, Joseph F. Arthur, Millicent N. K. Lamptey, Mohammed K. Abass, Amidu Gawusu, Francis Kumbel, Francis Osei-Yeboah, Linda Batsa Debrah, Dorcas O. Owusu, Alexander Debrah, Ertan Mayatepek, Julia Seyfarth, Richard O. Phillips, Marc Jacobsen

**Affiliations:** 1 Kumasi Centre for Collaborative Research in Tropical Medicine (KCCR), Kumasi, Ghana; 2 Department of General Pediatrics, Neonatology and Pediatric Cardiology, Medical Faculty, University Hospital Duesseldorf, Heinrich-Heine University, 40225 Duesseldorf, Germany; 3 Agogo Presbyterian Hospital, Agogo, Ghana; 4 St. Mathias Catholic Hospital, Yeji, Ghana; 5 Atebubu District Hospital, Atebubu, Ghana; 6 Sene West Health Directorate, Kwame Danso, Ghana; 7 School of Medicine and Dentistry, College of Health Sciences, Kwame Nkrumah University of Science and Technology (KNUST), Kumasi, Ghana

**Keywords:** Tuberculosis, Treatment response, Plasma cytokines

## Abstract

**Background:**

*Mycobacterium (M.) tuberculosis*-caused immunopathology is characterized by aberrant expression of plasma cytokines in human tuberculosis. Disease severity and long-term anti-mycobacterial treatment are potentially influenced by immunopathology and normalization of plasma cytokine levels during therapy may indicate treatment efficacy and recovery.

**Study design and methods:**

In this study, we analyzed the concentrations of selected plasma cytokines (i.e., IL-6, IP-10, IL-10, IL-22, IFNγ, GM-CSF, IL-8) and *M. tuberculosis* sputum burden in patients with tuberculosis (*n* = 76). Cytokine levels were compared to healthy contacts (*n* = 40) and changes under treatment were monitored (i.e., 6 and 16 weeks after treatment start). According to differences in *M. tuberculosis* sputum burden and conversion, tuberculosis patients were classified as paucibacillary as well as ‘rapid’ or ‘slow’ treatment responders. A subgroup of tuberculosis patients had fatal disease courses.

**Results:**

Six of seven cytokines were significantly higher in tuberculosis patients as compared to contacts and four of these (i.e., IL-6, IP-10, IL-10, and IL-22) were detectable in the majority of tuberculosis patients. IL-6 showed the strongest discriminating capacity for tuberculosis disease and in combination with IL-10 concentrations efficiently classified paucibacillary tuberculosis cases as well as those with fatal disease outcome. In addition, IL-6 and IP-10 levels decreased significantly after 6 weeks of treatment and analyses of subgroups with differential treatment response showed delayed decline of IL-6 levels in slow treatment responders.

**Conclusions:**

Combinations of different plasma cytokine (namely, IL-6, IL-10, and IP-10) efficiently classified tuberculosis patients with differential mycobacterial burden and especially IL-6 qualified as a biomarker candidate for early treatment response.

**Supplementary Information:**

The online version contains supplementary material available at 10.1007/s15010-022-01870-3.

## Introduction

Tuberculosis is a chronic infectious disease caused by *Mycobacterium (M.) tuberculosis*. Transmitted via aerosol to close contacts of tuberculosis patients, *M. tuberculosis* is controlled by host immune surveillance in the majority of individuals leading to asymptomatic latent infection. Key factors of immune protection (e.g., T helper type 1 cells, IFN-γ) cells are well known, but the exact mechanisms underlying tuberculosis disease progression——in 5–15% of adult individuals [1]—are not well defined. There is increasing evidence that tuberculosis pathology affects innate and adaptive immune cell phenotype and functions [2]. As a consequence, effector T-cell and antigen-presenting cell functions in tuberculosis patients are impaired with potential implications on anti-mycobacterial host response [3–5]. Importantly, several pathognomonic factors are detectable in blood and also affect immune cells, which had not been in direct contact with the pathogen itself or affected tissues [3–6]. This rendered systemic effects of *M. tuberculosis* caused disease likely. In accordance, several studies identified aberrant serum or plasma cytokine levels in patients with tuberculosis [5, 7–12]. For some of the altered cytokines, normalization of serum/plasma levels during anti-mycobacterial treatment of tuberculosis patients has been shown [7, 13–19]. Own previous studies provided evidence that aberrant high serum cytokines (i.e., IL-6, IL-10) in tuberculosis can affect T-cell functions, e.g., by causing constitutive STAT3 phosphorylation, in tuberculosis patients [8]. These findings prompted us to investigate candidate cytokine concentrations in tuberculosis patients with differential *M. tuberculosis* burden and disease course, contacts, as well as during therapy in well-characterized patient subgroups to identify plasma markers of disease severity and treatment response.

Diagnosis of tuberculosis is based on *M. tuberculosis* detection in sputum by smear microscopy and/or culture-based methods. Same methods are routinely applied to monitor treatment response of tuberculosis patients. In the present study, we analyzed tuberculosis patients by sputum smear and MGIT culture for mycobacteria detection prior to treatment, as well as at week 6, 9, 12 and 16. Based on this, tuberculosis patients were subdivided into paucibacillary (negative prior to treatment), rapid treatment responders (negative after 6 or 9 weeks), as well as slow responders (any positive test at or after week 6). Plasma cytokines were determined prior to treatment, after 6 and 16 weeks and concentrations were compared between subgroups and under treatment.

## Material and methods

### Study cohorts and clinical characterization

**Fig. 1:**
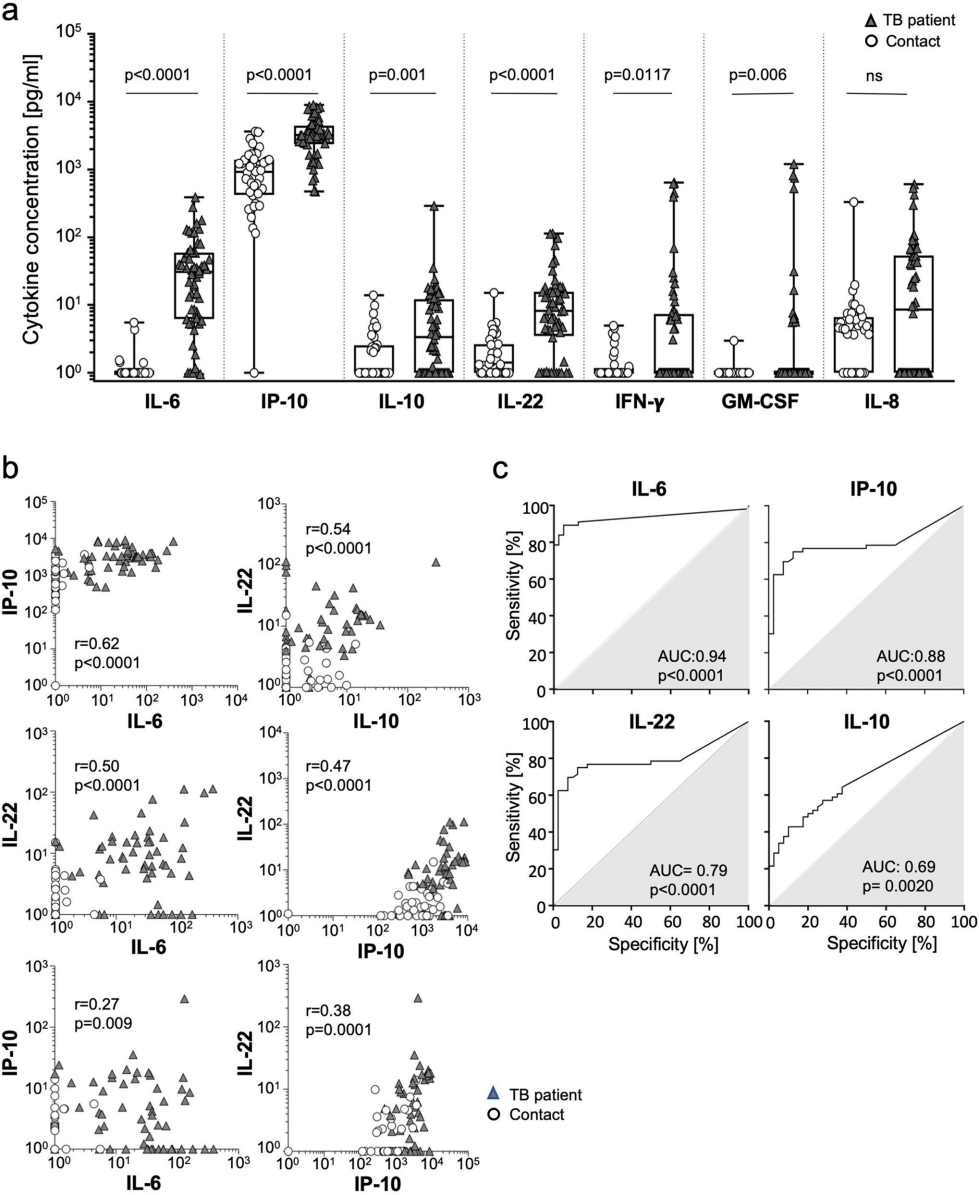
Candidate plasma cytokines for comparison and discrimination of tuberculosis patients and contacts*.* Seven selected cytokines (i.e., IL-6, IP-10, IL-10, IL-22, IFN-γ, GM-CSF, IL-8) were measured in plasma samples of confirmed tuberculosis patients and contacts. Comparisons of cytokines between the study groups (**a**) as well as a selected subset of cytokines for correlation (**b**) and discrimination of participants between the study groups (**c**) are depicted. Symbols indicate median values of duplicates measured for each tuberculosis patient (grey triangles) or AC (open circles). (**a**) A combined symbol/box graph depicts individual concentrations of indicated cytokines as well as study group distributions including median, 5, 25, 75, and 95 percentiles. Samples of tuberculosis patients (*n* = 56) and contacts (*n* = 40) have been included. The two-tailed Mann–Whitney *U* test was performed and nominal *p* values are given. A *p* value < 0.05 was considered significant. (**b**) Selected cytokines were analyzed for correlation in individual donors and are depicted as symbol plots. Samples of tuberculosis patients (*n* = 56) and contacts (*n* = 40) have been included. The Spearman Rank test was applied to determine significant correlations for all donors and both study groups separately. Correlation coefficients (*r*) and nominal *p* values are given. (**c**) ROC analyses for discrimination of tuberculosis patients (*n* = 56) and contacts (*n* = 40) using IL-6, IP-10, IL-22, and IL-10 plasma concentrations were performed. Graphs indicate sensitivity and specificity of classification as ROC curves. AUC as well as nominal *p* values are given. Contacts: household contacts; ROC: receiver operator characteristic; AUC: area under curve

**Fig. 2:**
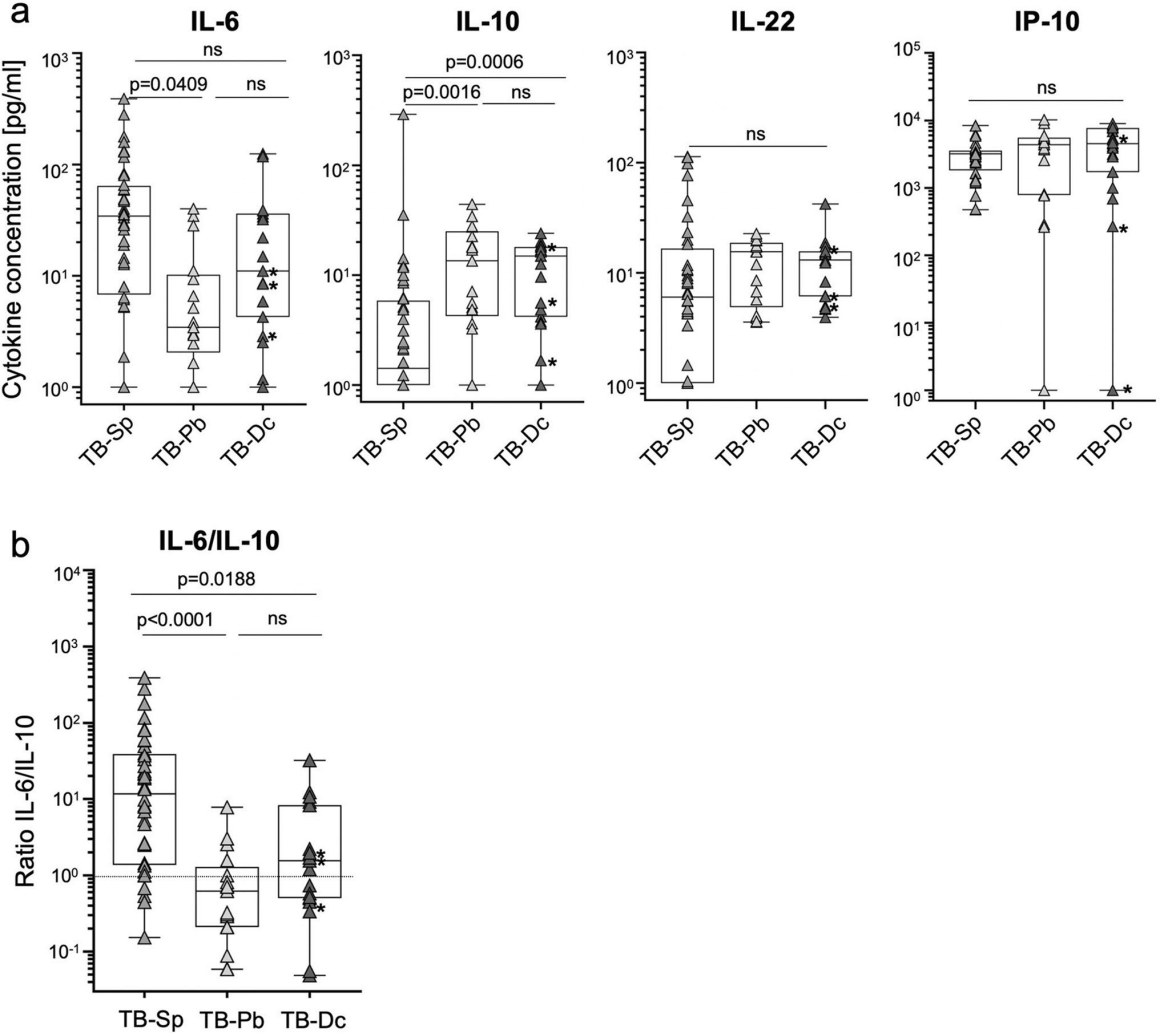
Tuberculosis patients’ subgroup comparison for IL-6, IP-10, IL-10, IL-22 concentrations and IL-6/IL-10 ratios. IL-6, IP-10, IL-10, and IL-22 plasma concentrations (**a**) and individual IL-6/IL-10 ratios (**b**) were compared between tuberculosis patients’ subgroups [i.e., *M. tuberculosis* sputum positive (TB-Sp; *n* = 40), *M. tuberculosis* sputum negative (‘paucibacillary’; TB-Pb; *n* = 17), and patients with a fatal disease course (‘deceased’; TB-Dc; *n* = 19)] in plasma samples of confirmed tuberculosis patients and contacts. Combined symbol/box graphs depict individual concentrations of indicated cytokines as well as study group distributions including median, 5, 25, 75, and 95 percentiles. Symbols indicate mean values of duplicates measured for each TB-Sp (dark gray triangles), TB-Pb (bright gray triangles), or TB-Dc (black triangles) patient. TB-Dc patients with paucibacillary manifestations (*n* = 3) are indicated by black color and asterisks. The two-tailed Mann–Whitney *U* test was performed and nominal *p *values are given. A *p* value < 0.05 was considered significant. (**b**) The dotted line indicates equal concentrations of IL-6 and IL-10

We recruited tuberculosis patients (*n* = 76) and household contacts of tuberculosis patients (contacts, *n* = 40) from April 2019 to September 2021 at the Agogo Presbyterian Hospital, the St Mathias Catholic Hospital, the Atebubu District Hospital, and the Sene West District Hospital. Diagnosis of active tuberculosis was based on patient history, clinical examination, chest X-ray, sputum smear and sputum culture-based tests. GeneXpert (Cepheid) analyses were done for all tuberculosis patients. Two patients with tuberculosis were HIV coinfected. All tuberculosis patients were included prior to initiation of treatment and blood was taken at baseline (BL), 6 weeks as well as 16 weeks thereafter. *M. tuberculosis* sputum samples were obtained at BL, 6 weeks, 9 weeks, 12 weeks, and 16 weeks. Analyses of *M. tuberculosis* sputum burden at BL formed the basis for classification of tuberculosis patients as sputum-positive (*n* = 56) or paucibacillary (*n* = 17) cases. In addition, sputum-positive patients at BL, who were successfully treated, were classified as ‘rapid’ (*n* = 20) or ‘slow’ (*n* = 20) treatment responders using sputum conversion. The exact criteria are given in Supplementary Figure 1. A subgroup of tuberculosis patients had a fatal disease course and died during treatment (*n* = 19; TB-Dc). Acute tuberculosis cases for the analyses (shown in Fig. 1) included all sputum-positive individuals (*n* = 56). TB-Dc were analyzed separately for subgroup comparisons (Fig. 2).

Contacts showed no symptoms of tuberculosis, but had close relatives living in the same household with indexed tuberculosis patients according to self-report and direct observation. Previous studies demonstrated that contacts recruited on the basis of these criteria had largely latent *M. tuberculosis* infection [4, 20]. Approximately, 90% of *M. tuberculosis*-infected individuals remain asymptomatic, but latently infected. Contacts were included as a control group to identify markers of immunopathology in acute tuberculosis. The study group details are summarized in Table 1. The present study received approval from the Committee on Human Research, Publication and Ethics (CHRPE/AP/023/18) at the School of Medicine and Dentistry at the Kwame Nkrumah University of Science and Technology (KNUST) in Kumasi, Ghana. All study subjects gave written informed consent prior to recruitment.

**Table 1:** Study group characteristics

Total Number (*n*)	TB-Sp^a^	TB-RR^b^	TB-SR^b^	TB-Pb	TB-Dc^c^	Contacts
	40	20	20	17	19	40
Age (mean, SD)	47.6 ± 14.6	47.9 ± 14.5	47.2 ± 14.7	44.8 ± 19.8	43.7 ± 15.2	46.1 ± 15.0
Males *n* (%)	25 (62.5)	12 (60)	13 (65)	13 (76.5)	15 (78.9)	25 (62.5)
HIV positive (*n*)	0	0	0	1	1	0
GeneXpert pos *n* (%)	34 (85)	17(85)	17 (85)	8 (47.1)	12 (63.2)	na
*Symptoms*						
Cough > 2 wks *n* (%)	38 (95.40)	18 (90)	20 (100)	9 (52.9)	15 (78.9)	na
Fever *n* (%)	12 (30.0)	6 (30)	6 (30)	3 (17.6)	3 (15.8)	na
Chest pain *n* (%)	31 (77.5)	15 (75)	16 (80)	8 (47.1)	9 (47.4)	na
Hemoptysis *n* (%)	6 (15.0)	4 (20)	2 (10)	3 (17.6)	1 (5.2)	na
Weight loss *n* (%)	23 (57.5)	9 (45)	14 (70)	5 (29.4)	8 (42.1)	na

TB-Sp, sputum-positive tuberculosis patients; TB-RR, tuberculosis patients with rapid treatment response; TB-SR, tuberculosis patients with slow treatment response; TB-Pb, paucibacillary tuberculosis patients; TB-Dc, deceased tuberculosis patients; wks, weeks. SD, standard deviation

^a^Acute tuberculosis patients (*n* = 56) comprised sputum-positive cases independent of treatment outcome (TB-Sp, *n* = 40; TB-Dc, *n* = 16)

^b^TB-RR and TB-SR are subsets of successfully treated TB-Sp patients

^c^Three TB-Dc were paucibacillary cases

### Quantification of plasma cytokines using cytometric bead assay

Frozen plasma samples were thawed overnight in the fridge at 4 °C. Biolegend LEGENDplex™ Multi-Analyte Flow Assay kit (Custom Human Assay) was used for the simultaneous detection of different cytokines (i.e., IL-6, IP-10, IL-22, IL-10, GM-CSF, IFNγ, IL-8) in plasma samples according to manufacturer’s instructions. Briefly, 12.5 µl of samples were diluted twofold in assay buffer and incubated with pre-mixed antibody-labeled beads (12.5 µl) for 2 h at room temperature. Thereafter detection antibody (12.5 µl) was added and incubated for 1 h. Finally, streptavidin–PE (12.5 µl) was added to samples and incubated for an additional 30 min. Samples were then washed and analyzed with a CytoFlex S flow cytometer (Beckman Coulter). Data were analyzed using the cloud version of the Biolegend LEGENDplex Data Analysis Software (Qognit. Inc). Concentrations were calculated using the respective cytokine standard. Values below the standard curve were termed undetectable and set to 1 pg/ml for depiction and calculations. For each cytokine, the proportion of tuberculosis patients with undetectable values (‘non-responders’) was calculated and only cytokines with less than 50% non-responders in the patients’ group were included for further analyses.

### Analyses and statistics

All statistical analyses were performed using GraphPad Prism v8 software (GraphPad Software, La Jolla CA, USA). Distribution tests (i.e., Kolmogorov–Smirnov, Shapiro–Wilk) did not suggest normal distribution, and, therefore, we used non-parametric tests throughout. Study group comparisons were performed by the Mann–Whitney *U* test. Spearman rank correlation was used to assess co-expression of cytokines. The Wilcoxon signed rank test was used for time course analysis. A *p* value below 0.05 was considered statistically significant. Graphs and heatmaps were generated using GraphPad Prism v8 and DISPLAYR software, respectively.

## Results

### Increased plasma cytokine concentrations in tuberculosis patients

Initially, we determined seven candidate cytokines in plasma samples from acute tuberculosis patients (*n* = 56) and household contacts (Contacts; *n* = 40). Six cytokines, i.e., IL-6, IP-10, IL-10, IL-22, IFN-γ, and GM-CSF, were significantly higher in plasma samples from patients with tuberculosis (Fig. 1a). IL-8 was not different between the study groups (Fig. 1a). IL-6, IP-10, IL-10, and IL-22 were detectable in the majority of tuberculosis patients (> 50%), whereas IFNγ and GM-CSF were measurable only in minor subsets of both study groups (IFNγ: 41.1% in patients, 25.0% in contacts; GM-CSF: 21.4% in patients, 2.5% in contacts). Hence, we focused on IL-6, IP-10, IL-10, and IL-22 for further analyses. Comparison of different cytokines in individual donors detected strong correlation between IL-6 and IP-10 (*r* = 0.62, *p* < 0.0001) as well as for IL-22 that showed strong correlation with IL-6 (*r* = 0.50, *p* < 0.0001), IL-10 (*r* = 0.54, *p* < 0.0001), and IP-10 (*r* = 0.47, *p* < 0.0001) (Fig. 1b). Moderate, but significant, correlations were detected between the other cytokines (Fig. 1b). These results identified concomitantly increased plasma cytokine candidates in patients with acute tuberculosis.

### IL-6 and IP-10 plasma concentrations discriminate tuberculosis patients from household contacts

Next, we analyzed the capacity of different cytokine candidates to discriminate tuberculosis patients from contacts. IL-6 was the most potent factor for discrimination in ROC analyses [area under the curve (AUC): 0.94, *p* < 0.0001]. IP-10 had also strong capacity to discriminate between the study groups (AUC: 0.88, *p* < 0.0001), whereas IL-22 (AUC: 0.79, *p* < 0.0001) and IL-10 (AUC: 0.69, *p* = 0.0020) were less efficient for discrimination (Fig. 1c). Combinations of different cytokines (i.e., sums) did not increase discriminatory power as compared to IL-6 alone (Supplementary Fig. 2). We concluded that single plasma cytokine levels, namely of IL-6 and IP-10, were most potent for classification of study participants. Next, we analyzed if *M. tuberculosis* sputum burden as a measure of disease severity was associated with plasma cytokine levels.

### Tuberculosis patients with paucibacillary disease manifestation have lower IL-6 and higher IL-10 plasma concentrations

*M. tuberculosis* sputum burden was determined and a subgroup of patients presented with negative sputum smear and culture prior to therapy (‘paucibacillary’; TB-Pb). These TB-Pb patients (*n* = 20) were not included in initial analyses, but were now compared to sputum-positive tuberculosis patients (TB-Sp; *n* = 40). In addition, we separated a subgroup of tuberculosis patients, whose disease outcome was death. (TB-Dc; *n* = 19). The majority of TB-Dc patients were part of the TB-Sp group (*n* = 16), but three TB-Dc patients were TB-Pb (Fig. 2; Table 1). No differences were detected for IP-10 and IL-22 between the subgroups (Fig. 2a). Lower IL-6 plasma levels were detected in TB-Pb as compared to TB-Sp patients (*p* = 0.049), whereas no IL-6 differences were seen for TB-Dc (Fig. 2a). Notably, IL-10 was significantly higher in TB-Pb and TB-Dc patients (*p* = 0.0016 and *p* = 0.0006, respectively) (Fig. 2a). Due to opposing results for IL-6 and IL-10, we calculated IL-6/IL10 ratios for individuals from all subgroups. IL-6/IL10 ratios were significantly higher in TB-Sp patients as compared to the TB-Pb (*p* < 0.0001) and TB-Dc patients (*p* = 0.017; Fig. 2b). Actually, similar IL-6 and IL-10 plasma levels were detected in TB-Pb, whereas median levels for IL-6 were approximately tenfold higher than for IL-10 in TB-Sp patients (Fig. 2b). Marked differences in concomitant IL-6 and IL-10 levels between subgroups prompted us to determine the discriminatory power of different factors. IL-6 alone showed discriminatory capacity between TB-Sp and TB-Pb (AUC: 0.79, *p* = 0.0003), and IL-10 was effective for discrimination of TB-Sp and TB-Dc (AUC: 0.82, *p* < 0.0001) Table 2. Notably, IL-6/IL-10 ratios showed strongest capacity to discriminate TB-Sp and TB-Pb (AUC: 0.88, *p* < 0.0001), suggesting that opposing IL-6 and IL-10 levels characterize these tuberculosis patient subgroups (Table 2).

**Table 2:** Receiver operating characteristic (ROC) analyses of tuberculosis patient subgroups

Analyte	Comparisons	AUC	*p* value
IL-6	TB-Sp versus TB-Pb	0.80	0.0004
	TB-Sp versus TB-Dc	0.63	0.0980
**IL-10**	TB-Sp versus TB-Pb	0.79	0.0007
	**TB-Sp versus TB-Dc**	**0.82**	** < 0.0001**
**IL-6/IL-10**	**TB-Sp versus TB-Pb**	**0.88**	** < 0.0001**
	TB-Sp vs TB-Dc	0.76	0.0014

Optimal discrimination factors/calculations between TB-Sp and TB-DC or TB-Pb are indicated by bold font

TB-Sp: *n* = 40; TB-Pb: *n* = 17; TB-Dc: *n* = 19; IL-6, Interleukin-6; IL-10, Interleukin-10; ROC, receiver operating characteristic; TB-Sp, sputum-positive tuberculosis patients; TB-Pb, paucibacillary tuberculosis patients; TB-Dc, deceased tuberculosis patients

### IL-6 and IP-10 plasma concentrations decline during anti-mycobacterial treatment of tuberculosis patients

**Fig. 3:**
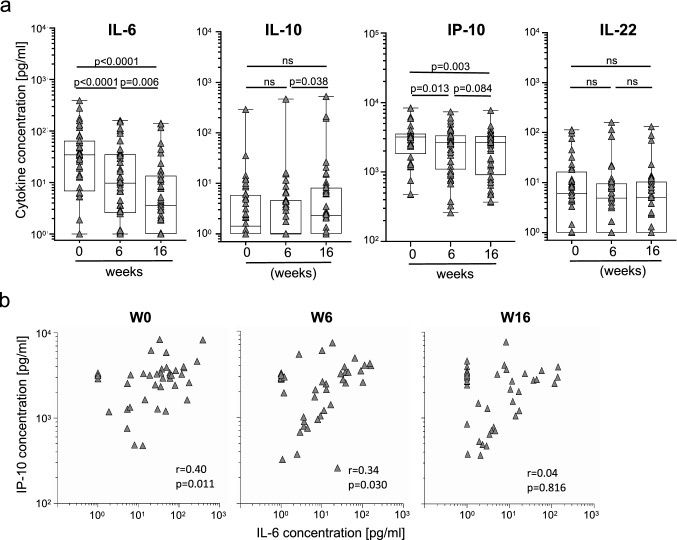
Comparison of tuberculosis patients before and under treatment for IL-6, IP-10, IL-10, IL-22 plasma concentrations. (**a**) IL-6, IP-10, IL-10, and IL-22 plasma concentrations were compared before and during treatment of tuberculosis patients’ subgroups (*n* = 40) (i.e., prior to treatment ‘W0’; 6 weeks ‘W6’ and 16 weeks ‘W16’ after treatment start). Combined symbol/box graphs depict individual concentrations of indicated cytokines as well as study group distributions including median, 5, 25, 75, and 95 percentiles. The Wilcoxon signed rank test was performed and nominal *p* values are given. A *p* value < 0.05 was considered significant. (**b**) Correlation of IL-6 and IP-10 plasma concentrations are shown for each time point during treatment. Dark grey triangles indicate median values of duplicates measured for each patient on W0, W6, or W16. The Spearman rank test was applied to determine significant correlations for all donors and both study groups separately. Correlation coefficients (*r*) and nominal *p* values are given

Next, plasma samples of TB-Sp patients were compared prior to treatment and at follow-up (i.e., 6 and 16 weeks after treatment start). IL-10 and IL-22 concentrations showed no significant decline during treatment and IL-10 even increased between week 6 and 16 (Fig. 3a). In contrast, IL-6 and IP-10 concentrations declined during treatment (*p* < 0.0001, *p* = 0.003, respectively) (Fig. 3a). Both, IL-6 and IP-10, showed decreased concentrations at week 6 (*p* < 0.0001, *p* = 0.013, respectively) and IL-6 further declined between week 6 and 16 (*p* = 0.006; Fig. 3a). Since IL-6 and IP-10 plasma concentrations correlated prior to treatment (Fig. 1b), we next analyzed correlation during treatment course. IL-6 and IP-10 correlated in tuberculosis patients prior to treatment (*r* = 0.4, *p* = 0.011) and at week 6 (*r* = 0.34, *p* = 0.030). However, the correlation between IL-6 and IP-10 vanished until week 16 (*r* = 0.03, *p* = 0.84). These results suggested differences in time courses between plasma cytokines during treatment and identified IL-6 and IP-10 as an early marker of treatment response in tuberculosis patients.

### Rapid and slow treatment responders differ in decline of IL-6 plasma concentrations between week 6 and 16

**Fig. 4:**
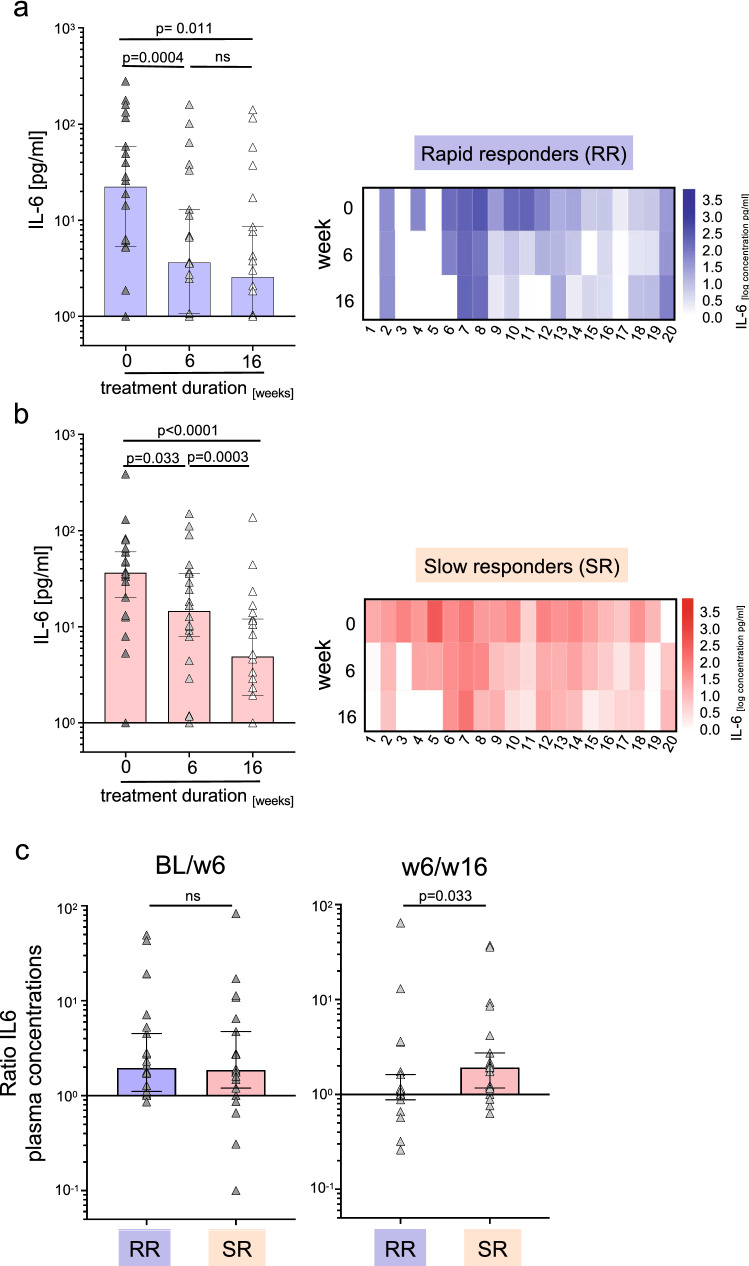
IL-6 plasma levels of tuberculosis patients classified as ‘rapid’ or ‘slow’ responders during treatment. IL-6 plasma concentrations were compared before and during treatment (i.e., prior to treatment ‘W0’; 6 weeks ‘W6’ and 16 weeks ‘W16’ after treatment start) in tuberculosis patients’ subgroups classified as ‘rapid’ (blue background; *n* = 20) or ‘slow’ (red background; *n* = 20) treatment responders (for details see Methods section and Supplementary Fig. 1). Combined bar/symbol graphs (**a**) and heat maps (**b**) depict individual concentrations of IL-6. (**a, c**) Bar/symbol graphs depict study group distributions including median and 95% confidence interval. (**c**) Calculated ratios of IL-6 plasma concentrations between W0 and W6 as well as W6 and W16 are shown as bar/symbol graphs and compared between both subgroups. Dark gray triangles indicate median values of duplicates measured for each patient on W0, W6, or W16. The Wilcoxon signed rank test was performed and nominal *p* values are given. A *p* value < 0.05 was considered significant. BL, baseline, before treatment start; w6, 6 weeks after treatment start; w16, 16 weeks after treatment start; RR, rapid treatment responders; SR, slow treatment responders

Treatment efficacy in tuberculosis patients can be assessed by *M. tuberculosis* detection in sputum samples. The samples were analyzed at baseline as well as 6, 9, 12, and 16 weeks after treatment initiation. TB-Sp patients with negative sputum samples already after 6 or 9 weeks were classified as ‘rapid’ treatment responders, whilst those with any positive test after week 6 were classified as ‘slow’ treatment responders (Supplementary Figure 1). Rapid and slow responders showed no differences in IL-6, IP-10, IL-22, and IL-10 plasma concentrations prior to treatment (Supplementary Figure 3a). Time course measurements detected no differences for IL-22 and IL-10 during treatment for both rapid and slow responders and IP-10 showed a significant decrease for the slow responder group (*p* = 0.002; Supplementary Figure 3b). Notably, rapid and slow responders differed in IL-6 changes during treatment (Fig. 4a, b). Rapid responders had significantly declined IL-6 concentrations at week 6 (*p* = 0.004) and no differences between week 6 and 16 (Fig. 4a). In contrast, slow responders had significantly declined IL-6 plasma concentrations also between week 6 and 16 (*p* = 0.0003) (Fig. 4b). To compare individual IL-6 changes between BL and week 6 as well as week 6 and week 16 for rapid and slow responders, we calculated fold-changes (i.e., BL vs. week 6; week 6 vs. week 16) for each individual donor. Study group comparisons detected similar fold-changes between week 0 and week 6 (median fold-change RR: 1.97; SR: 1.87; *p* = 0.799; Fig. 4c), but only slow responders showed increased fold-changes between week 6 and 16 (median fold-change RR: 1.012; SR: 1.931; *p* = 0.033; Fig. 4c). We concluded that although the interindividual variability in initial IL-6 levels and changes was marked (Fig. 4a, b), study groups of rapid and slow responders differed in the time interval of IL-6 normalization. Whereas IL-6 plasma levels normalized in rapid responders within the first 6 weeks, this process was still ongoing between week 6 and 16 in slow responders.

## Discussion

Biomarkers of treatment efficacy are urgently needed in tuberculosis to shorten long-term treatment [21, 22]. Since detection of *M. tuberculosis* in sputum smear and culture—the gold standard for monitoring treatment efficacy—are hampered by limited sensitivity and frequent culture contamination, immune markers can help in treatment monitoring. This study investigated plasma cytokine levels in acute tuberculosis and during treatment. Six of seven analyzed cytokines showed increased plasma concentrations in acute tuberculosis. However, only four cytokines (i.e., IL-6, IP-10, IL-22, IL-10) were detectable in the majority of patients. Higher IL-6 levels discriminated efficiently between tuberculosis patients and contacts. In addition, IL-6 concentrations declined during anti-mycobacterial treatment and patients with early sputum conversion showed more rapid IL-6 normalization as compared to slow treatment responders. Hence, IL-6 qualifies as a candidate marker for treatment efficacy and recovery in tuberculosis patients. These results were in accordance with previous studies, which found increased IL-6 plasma levels in tuberculosis patients [7–9, 11, 12, 23] and normalization during treatment [7, 11, 24].

IL-6 plasma concentration may also be a marker of disease severity since we detected lower IL-6 levels in tuberculosis patients with low *M. tuberculosis* sputum burden (TB-Pb). This finding is controversial since others did not detect an association of higher IL-6 plasma concentrations with mycobacterial sputum burden or disease severity [7, 11]. Heterogeneity of tuberculosis disease [25] as well as marked interindividual variability—as also seen in the present study—may account for these differences. In addition, our study suggested that concomitant analyses of different cytokines may better reflect study group differences. We demonstrated that IL-10 plasma levels were higher in TB-Pb patients, whereas IL-6 was lower as compared to TB-Sp patients. We as well as others found higher expression of IL-10 in tuberculosis patients [7, 8, 24]. IL-10 is a key cytokine of immune regulation and has anti-inflammatory effects [26]. Although the exact role of IL-10 (and IL-10 family cytokines) is not clear yet [27], a role of immune regulation and the relevance of inflammatory as well as anti-inflammatory mechanisms in tuberculosis is well established [28]. Here, we found that IL-6/IL-10 ratios were optimal for discrimination between TB-Pb and TB-Sp patients. Previously, we demonstrated that aberrant high serum IL-6 and IL-10 concentrations affected T-cell functions in tuberculosis patients [8]. Association IL-6/IL-10 with high constitutive STAT3 phosphorylation was found in T cells of acute tuberculosis patients and impaired effector functions of *M. tuberculosis* specific T cells were detected [8]. These and other changes in tuberculosis resemble common effects of chronic inflammation on host immune response [2]. A key player in these processes is SOCS3, an inhibitor of IL-6 (but not IL-10) induced STAT3 signaling [29, 30]. A central role of SOCS3 in tuberculosis is strongly suggested [31, 32]. Against this background we consider it likely that changes in inflammatory/anti-inflammatory cytokines as well as the relative expression in plasma, e.g., IL-6/IL-10 ratios, may affect host immune functions. Therefore, a decrease in IL-6 plasma concentrations under treatment may also indicate recovery from immunopathology during active tuberculosis. Moreover, since immunopathology also impairs generation of T-cell memory [5, 8], we hypothesize that plasma cytokine normalization is crucial for generation of *M. tuberculosis* specific memory in tuberculosis patients to avoid recurrent disease.

In conclusion, we observed that a combination of IL-6, IL-10 and IP-10 could potentially differentiate between tuberculosis patients with different mycobacterial burden. Furthermore, IL-6 was identified as a marker of early treatment response in tuberculosis patients and holds promise for treatment monitoring. Interindividual variability and potential differences between tuberculosis patients globally limit the applicability of plasma cytokine concentrations as diagnostic markers or biomarkers of treatment. Hence, a combination of different marker sets (e.g., plasma candidates, in vitro expressed cytokines induced by antigen-specific stimulation) as well as adjusted thresholds may finally be used in clinical routine.

## Supplementary Information

Below is the link to the electronic supplementary material.

Supplementary file1 (PDF 2487 KB)
