# Supplementary material for: Plasma cytokine levels characterize disease pathogenesis and treatment response in tuberculosis patients
Source: Infection. 2022 Jun 27;51(1):169–79. doi: 10.1007/s15010-022-01870-3 (PMC9879809; doi:10.1007/s15010-022-01870-3)
Supplement: Supplementary file 1 — Supplementary file1 (PDF 2487 KB) [file 15010_2022_1870_MOESM1_ESM.pdf]

Supplementary Figure 1

Recruitment scheme and classification

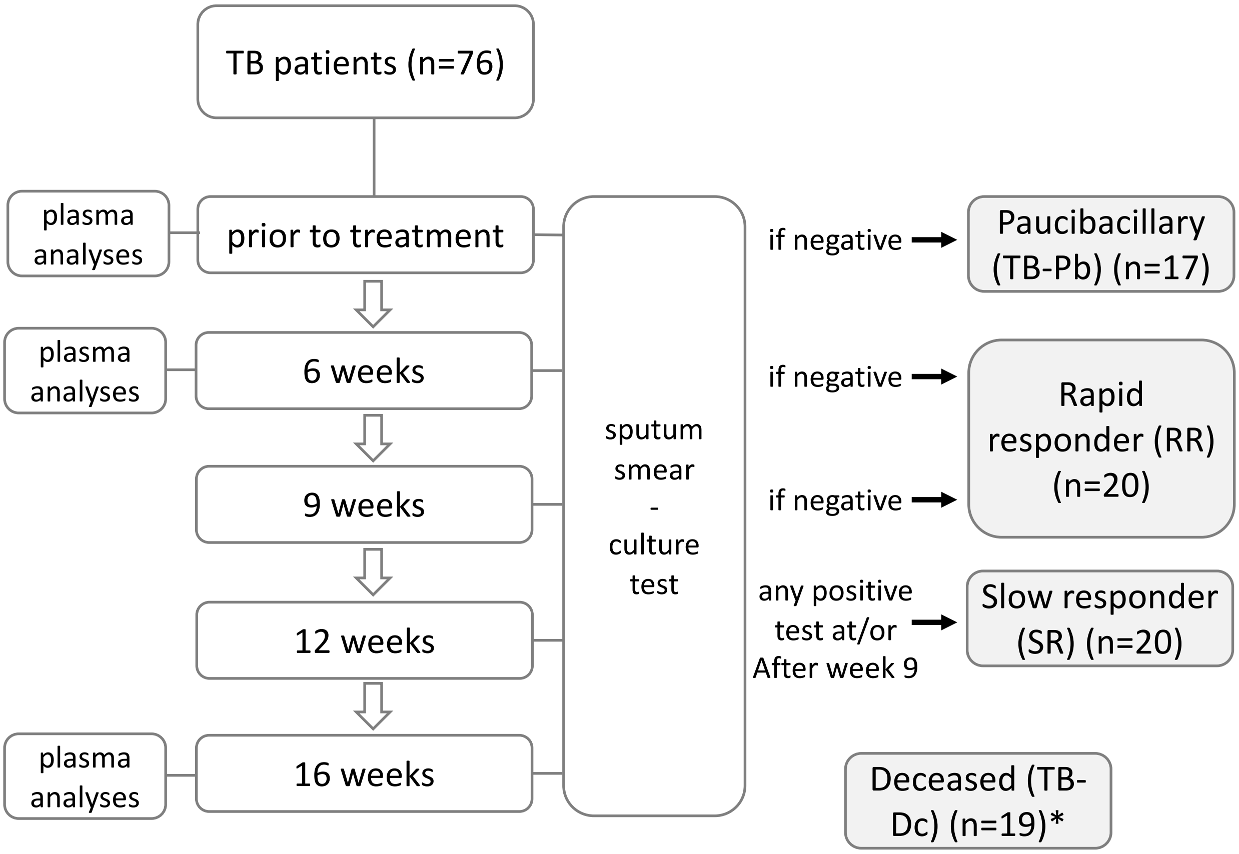

Recruitment scheme for plasma cytokine and sputum analyses as well as criteria for classification of tuberculosis patient subgroups. * Three TB-Dc patients were paucibacillary according to criteria (see Methods). TB-Sp, sputum positive tuberculosis patients; TB-Pb, paucibacillary Tuberculosis patients; TB-Dc, deceased tuberculosis patients.

Supplementary Figure 2

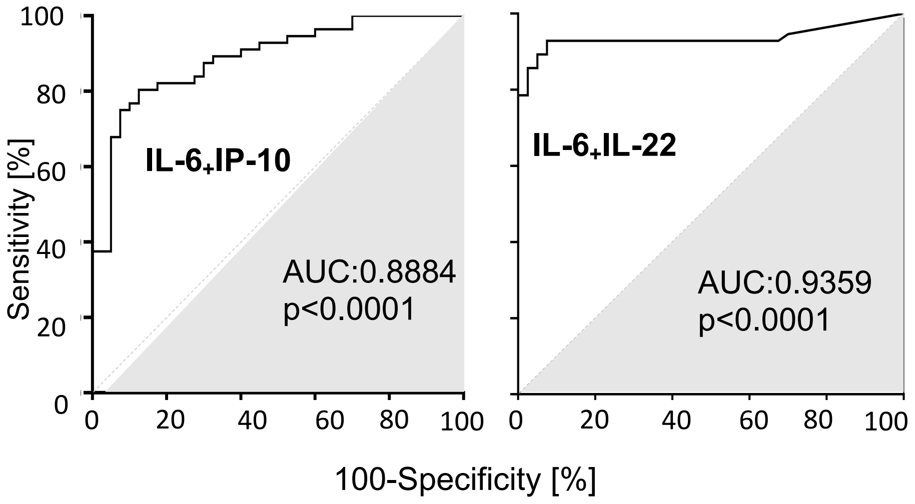

Receiver Operator Characteristic analyses for discrimination of tuberculosis patients and Controls using summed values for IL-6,IP-10 as well as IL-6,IL-22 plasma concentrations were performed. Graphs indicate sensitivity and specificity of classification as ROC curves. AUC as well as nominal p-values are given. Controls: asymptomatic household contacts; AUC: Area Under Curve.

Supplementary Figure 3

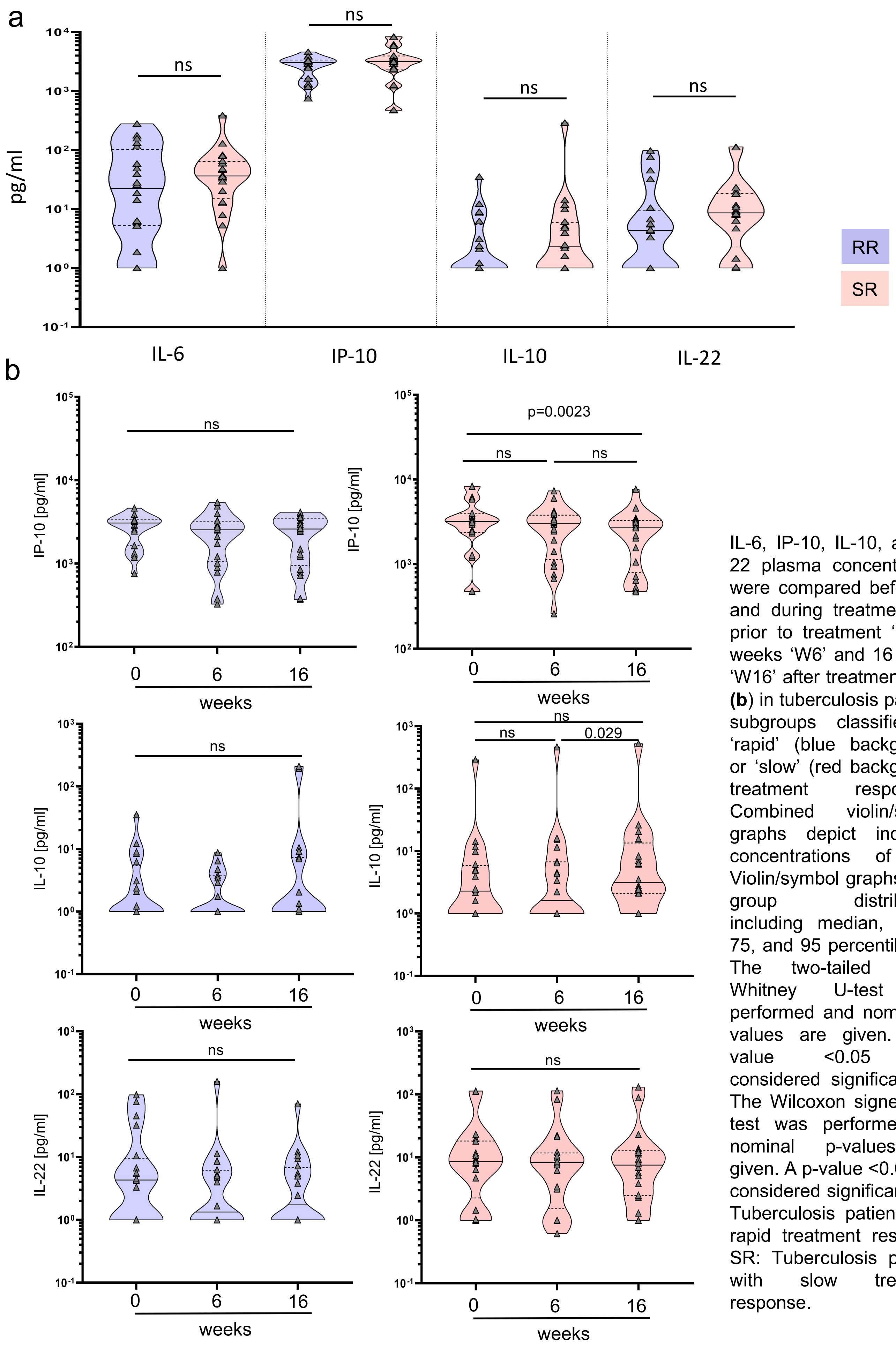

IL-6, IP-10, IL-10, and IL-22 plasma concentrations were compared before (**a**) and during treatment (i.e, prior to treatment 'W0'; 6 weeks 'W6' and 16 weeks 'W16' after treatment start) (**b**) in tuberculosis patients' subgroups classified as 'rapid' (blue background) or 'slow' (red background) treatment responders. Combined violin/symbol graphs depict individual concentrations of IL-6. Violin/symbol graphs study group distributions including median, 5, 25, 75, and 95 percentiles. (**a**) The two-tailed Mann-Whitney U-test was performed and nominal p-values are given. A p-value <0.05 was considered significant. (**b**) The Wilcoxon signed rank test was performed and nominal p-values are given. A p-value <0.05 was considered significant. RR: Tuberculosis patients with rapid treatment response; SR: Tuberculosis patients with slow treatment response.
